# Mytilicola orientalis

**DOI:** 10.1007/s10499-022-00928-1

**Published:** 2022-07-16

**Authors:** Yannick Borkens, Paul Koppe

**Affiliations:** 1 College of Public Health, Medical and Veterinary Science, James Cook University, Townsville, QLD Australia; 2 College of Science and Engineering, James Cook University, Townsville, QLD Australia

**Keywords:** Aquatic and veterinary parasitology, Copepods, Mussels, *Mytilicola orientalis*, Neozoa, Shellfish aquaculture

## Abstract

Neozoa are invasive species that enter faunal communities as new species. Not infrequently, they pose a threat to local ecosystems. Climate change could further promote these developments or favor neozoa. Thus, they represent a relevant threat in the future. One of these neozoa is the copepod parasite *Mytilicola orientalis*. This parasite originates from Asia and infects a wide variety of bivalves like mussels and oysters. However, as an invasive species, it can be found more and more frequently in Europe, especially in the North and Baltic Seas. There, *M*. *orientalis* poses a real threat to mussels in aquaculture and thus also to the local economy.

## Introduction

Parasites are highly specialized organisms that infest hosts such as animals, plants, or fungi. Mostly, body fluids of the hosts serve the parasite as food and resource. In most cases, the evolution of the parasites is closely adapted to that of the hosts. This is referred to as antagonistic coevolution (Paterson et al. 2010). Parasites are not a unified taxon, but include many different species of the different taxonomic groups, for example, protozoa, helminths, or insects. A distinction is made between endo- and ectoparasites (rb 2015). Parasites do not only represent a relevant medical hazard for humans as well as for domestic and farm animals. As veterinary pathogens, parasites also pose a substantial threat to agriculture and other economic sectors (Mennerat et al. 2010).

**Fig. 1:**
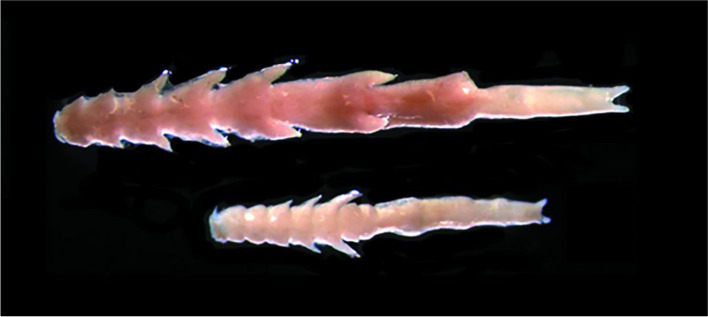
Image of *Mytilicola orientalis*. A female is shown above, a male below (Goedknegt et al., 2018)

**Fig. 2:**
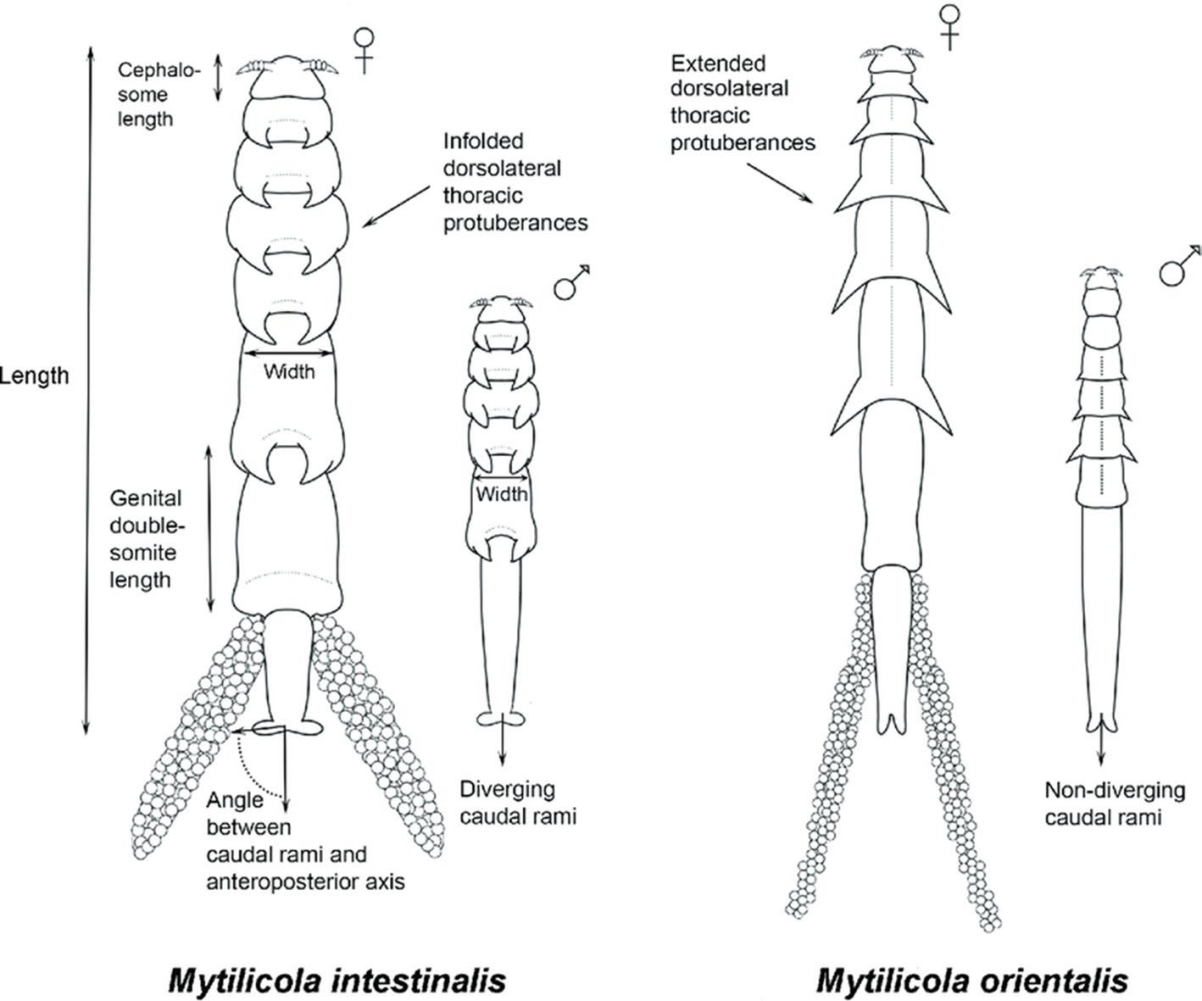
Schematic comparison of *Mytilicola intestinalis* and *Mytilicola orientalis*. Both sexes are shown (Goedknegt et al., 2018)

The *Mytilicola* group includes copepod parasites that infest various bivalve species. The copepods have been detected in the Adriatic Sea (Kovačić et al. 2018) and the North Sea (Thieltges et al. 2008), among others, but also in America. Reports from America come from Alaska (Alaska Center for Conservation Science 2017), British Columbia (Bernard 2011), and the San Francisco Bay Area (Bradley and Siebert 1978). The first evidence comes from the Gulf of Trieste. Later records are from Marseille, Cuxhaven, and Northumberland (Hochley 1951; Yılmaz et al. 2020). Compared to its close relative *M*. *intestinalis*, *M*. *orientalis* is much more slender. In both species, the female (circa 8 mm) is significantly larger than the male (circa 4 mm). The parasite is shown in both Fig. 1 and Fig. 2. Figure 1 shows a photo of *M*. *orientalis*. Figure 2 compares *M*. *orientalis* and *M*. *intestinalis*.

The eggs of *Mytilicola* spp. hatch into nauplii 0.2 mm in size. These nauplii develop into cyclopids that already have mandibles and maxillae as well as abundant bristled pairs of feet. At this stage, the parasites infest their mollusc hosts. Inside the hosts, they grow into adults. During this development, both bristle occupation and abdominal articulation are reduced. As an adult, *Mytilicola* lives in the host intestine. There, the parasites move by alternately spreading and retracting pairs of legs and dorsal thoracic cusps (Steuer 1902).

Histochemical examination of the gut contents of *M*. *intestinalis* observed in situ revealed that the diet of the copepod is primarily herbivorous. This suggests that the parasite feeds on the intestinal content of the host and not on host tissue (Moore et al. 1978).

## Taxonomy

*Mytilicola* spp. was first described in 1902 by the Austrian biologist and zoologist Adolf Steuer. He was able to isolate a red-colored copepod from mussels of the Trieste Gulf. Dr. Steuer described that almost every mussel examined was infected with this copepod and assigned the new species to the genus *Dichelestiidae* (Steuer 1902). The name *Mytilicola* is derived from the order Mytilida. These include, among other representatives of the *Bivalvia*, the mussels. *Mytilicola intestinalis* is the type species.

*Mytilicola orientalis* was described by Mori in Japan in 1935. The species *Mytilicola ostreae*, described in 1938, is now considered a synonym of *M*. *orientalis* (Mori 1935).

Nowadays, the genus *Mytilicola* is classified in the family Mytilicolidae. In addition to *Mytilicola* spp., this also includes the genera *Cerastocheres*, *Pectonophilus*, and *Trochicola*. As with *Mytilicola* spp., these genera are parasites. The complete systematics of *Mytilicola orientalis* is:

Domain:    Eukaryota

Kingdom:  Metazoa

Phylum:    Arthropoda

Subphylum: Crustacea

Superclass:Multicrustacea

Class:       Hexanauplia

Subclass:  Copepoda

Order:     Poecilostomatoida

Family:   Mytilicolidae

*Genus:    Mytilicola*

*Species:  Mytilicola orientalis*

## Host

Hosts for *M*. *orientalis* are various mussel species. In the northern Wadden Sea, for example, the native mussel *Mytilus eduli*s (common mussel) shows a heavy infestation (Stock 1993). Based on the descriptions in the first records, it can be assumed that the parasite has become well established in the local population (Dethlefsen 1972). The European oyster (*Ostrea edulis*) also exhibits heavy infestation (Elsner et al. 2011). The Pacific oyster (*Crassostrea gigas*) represents a special case. This Pacific species is itself an invasive species that became established in Mussel Beds in the European Wadden Sea in the 1990s. Here it became an integral part of the biotic community. As an epibiont, it lives on densely packed mussel beds (Reise 1998). *M*. *orientalis* was most likely introduced to Europe with *C*. *gigas* (Stock 1993). Here, the parasite quickly transmitted to *O*. *edulis* and *M*. *edulis*. On the other hand, *M*. *intestinalis* could not be detected in *C*. *gigas* (Elsner et al. 2011). It is likely that *M*. *orientalis* will become the dominant *Mytilicola* species in Europe.

## Disease

*Mytilicola orientalis* causes the so-called red worm disease. Since larger mussels harbor more parasites, there is a positive correlation between the size of the host and the intensity of infestation. Juvenile mussels with less than 10 mm in length are rarely infected (Williams 1967). Due to the short life span of *M*. *orientalis*, an accumulation between the parasite and increased host age is unlikely (Davey and Gee 1976). The increased number of parasites in larger hosts may be due to the higher filtration rate of the mussels (Davey and Gee 1976).

The effect of infection on the host is subject of intense debate. While studies from the 1960s held *Mytilicola intestinalis* responsible for mass mortalities in various populations, later studies criticized these results and found fault with the statistical analysis. It has been shown that in the studies criticized, some mussels were not examined at all for the presence of *M. intestinalis*. For this reason, other sources like environmental factors may cause the deaths. Some findings of the older studies could not be reproduced more recent (Dollfus 1951; Davey and Gee 1988).

Infections with *Mytilicola* cause local metaplastic changes in the intestinal epithelium. Among these changes is the replacement of normal ciliated columnar cells by nonciliated cuboidal cells. Accumulation of hemocytes, which was expected as a common response of mussels in such disease, is not evident (Moore et al. 1978; Robledo et al. 1994). However, limited hemocytic infiltration into the intestinal epithelium and surrounding connective tissue has been noted. This infiltration occurs in the vicinity of the copepod (Figueras et al. 1991; Villalba et al. 1997). The role of the different developmental stages of *Mytilicola* and their influence on disease manifestation is also controversial. In the 1970s, Campbell postulated that the juvenile stages do most of the damage to the host, as they were partially detected in the hepatopancreas (Campbell 1970). Moore and colleagues later again found no evidence of this pathology. According to them, the few copepods that invaded and attached to the tubule epithelium were encapsulated and killed. The resulting damage to host tissues healed rapidly with no significant effect on basic cellular function (Moore et al. 1978). Deaths of mussels can be attributed to mechanical blockages of the mussel gut. These occur at high concentrations of *Mytilicola*. This density-dependent mortality probably occurs during the development of *Mytilicola* (Gee and Davey 1986).

Because *Mytilicola* spp. infect bivalves, the parasites pose no threat to humans. They simply cannot become infected with *Mytilicola*. Consumption of infested mussels is safe. The danger posed by *Mytilicola* to humans is therefore purely of an economic nature.

## Implications for shellfish aquaculture in a changing marine environment

The terms *aquaculture* and *fish farming* are often used interchangeably, but that would be like using *agriculture* and *cattle farming* interchangeably. While *fish farming* (or pisciculture) describes the commercial breeding of fish in fish tanks or fish ponds, usually for food, *aquaculture* is the entire process of producing aquatic animals and plants (mostly but not only for food) (Lucas 2015). In fact, finfish account for less than half of global aquaculture production by weight (54.3 million metric tons), with a 2018 farmgate value of 129.7 billion. The remaining production volume is accounted by aquatic algae (32.4 million metric tons), molluscs (17.7 million metric tons), crustaceans (9.4 million metric tons), and others (< 1 million metric tons), with a total annual aquaculture value of US$263.6 billion. In 2018, aquaculture overtook global wild-caught fish production for human consumption with 52% and 46% of total aquaculture production (Morro et al. 2021). In 2020, sales of aquaculture products and services decreased by 16.8% due to the COVID-19 pandemic (van Stenten et al. 2020; Rocha et al. 2022).^1^

**Fig. 3:**
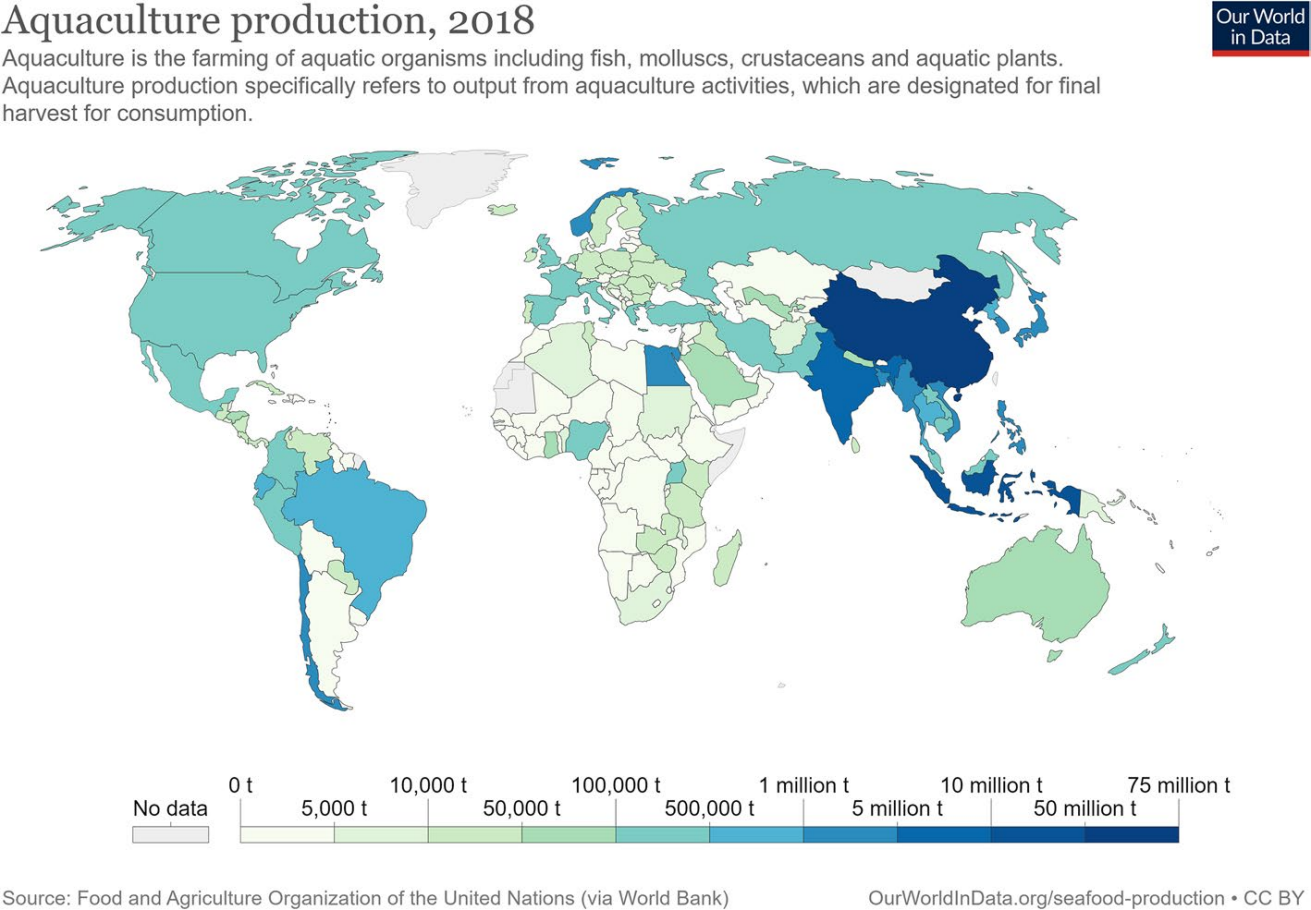
Aquaculture production in 2018. The map was derived from *Our World in Data*. https://ourworldindata.org/fish-and-overfishing

However, the cultivation of fish and other aquatic life did not originate in recent years. The history of aquaculture dates back to 1000 BC. At that time, primitive aquaculture techniques were used in ancient China to raise wild-caught carp. Nevertheless, aquaculture is now often referred to as a young industry because of its recent commercialization and increase in production volume. In just 17 years, global production of forage fish tripled (Naylor et al. 2021). Production of aquatic plants and algae also tripled during this period. Global production of farmed molluscs grew at an annual rate of 3.5%, lower than that of farmed fish (5.7%) and crustaceans (9.9%) (Tacon 2019; Naylor et al. 2021). Based on these data, aquaculture is currently ranked as one of the fastest growing industries globally, with a current growth rate of 5.8% and accounting for more than half of global seafood production. With 63,700,000 tons, most aquaculture products come from the People´s Republic of China. This is followed by Indonesia (16,600,000 tons) and India (5,703,002 tons). With 1,326,216 tons, Norway ranks first among European countries (9th on the international list). In general, aquaculture has a long history in Northern Europe. Already by the 1850s, early aquaculture had been established in Norway. Today, *Salmo salar* (the Atlantic salmon; Norway and Faroe Islands), *Oncorhynchus mykiss* (the rainbow trout; Denmark, Finland, and Sweden), *Salvelinus alpinus* (the Arctic char; Iceland), and *Gadus morhua* (the cod; Iceland) are the dominant cultured fish (Paisley et al. 2010). The map presented in Fig. 3 shows the global distribution of aquaculture production in 2018.

Reportedly, 62% of edible fish will come from aquaculture by 2030. Total fish supply will increase from 154 million tons in 2011 to 186 million tons in 2030. The fastest growth in aquaculture is expected for tilapia and shrimp (Kobayashi et al. 2015). This development is likely to be accelerated by climate change. Climate change poses a significant threat to the international food supply. Of course, aquaculture is also strongly affected by it. On the one hand, it affects the production rate, and on the other hand, it also affects sustainability (Maulu et al. 2021). The industry´s sustainability will be affected as increased temperatures negatively affect cold-water aquaculture production rate, namely by increasing mortality rates. Similarly, ocean acidification caused by rising temperatures will increase the likelihood of red tides causing significant losses and management costs (Maulu et al. 2021). However, aquacultures also have an important potential. Due to ecosystem change as well as warming, a lot of agricultural land will be lost in the future. It is estimated that 10 million hectares of arable land will be lost each year. Thus, it will become increasingly difficult to provide people with sufficient food in the future (Haberl et al. 2011; Borrelli et al. 2020). Hazards such as overfishing as well as microplastic pollution add to this problem (Shim and Thompson 2015; Ferguson-Cradler 2021). Among other methods, such as genetic engineering, aquacultures and selective breeding of certain species are a possible solution (Yazdi and Shakouri 2010; Kovak et al. 2022). We are not only talking about fish species, but specifically aquatic plants and algae. In addition, crustaceans, insects, and molluscs represent possible meat alternatives (Elhassan et al. 2019; de Carvalho et al. 2020). However, like fish, alternatives such as molluscs and arthropods are also threatened by emerging diseases and neobiota. Besides the destruction of important living and agricultural space, the increase of zoonotic diseases and neobiota represent one of the most relevant threats of climate change. Due to their relevance to food production and the associated economy, aquaculture already plays an important role within veterinary medicine (Meyer 1991). Pathogens and diseases can threaten the living organisms in aquaculture and thus posing a risk to animal health as well as the economy behind them. *M*. *orientalis* is only one example. Other examples are fish lice, *Streptococcus* spp., *Vibrio* spp., or even herpes viruses (Tengs and Rimstad 2017).

In the future, the warming of temperate climates will make it easier for invasive species to gain a foothold and spread in these regions. Because they often lack local predators in new biotopes, they pose a serious hazard to the biosphere. Climate change is thought to be driving the spread of many invasive species (Bernanke and Köhler 2009; Robinson et al. 2020). *M*. *orientalis* is likely to benefit from these changes and continue to spread in Europe (Meyer and Mann 1950; Reise 1998). To respond appropriately to this threat, experts need to take these neobiotes seriously and acquire further knowledge about them. However, due to a lower relevance or lack of medical danger to humans, these pathogens could be ignored. It would be a dangerous development if neobiotes such as *Mytilicola orientalis* were given the status of a neglected disease or neglected pathogen. After all, recent evidence, including the further emergence of concepts such as One Health, shows that human health is closely linked to the health of the planet and the environment.

## Data Availability

Not applicable.

## Footnotes

1 For more information, see *The State of World Fisheries and Aquaculture-Report*. The 2021 report will likely be released later this summer. The 2020 report is available online. https://www.fao.org/3/ca9229en/ca9229en.pdf
